# Supplementary material for: Increased alertness and moderate ingroup cohesion in bonobos’ response to outgroup cues
Source: PLoS One. 2024 Aug 21;19(8):e0307975. doi: 10.1371/journal.pone.0307975 (PMC11338468; doi:10.1371/journal.pone.0307975)
Supplement: S1 File — (ZIP) [file pone.0307975.s001.zip › Final/StabilityandColinearitySupporting.docx]

For all of our GLMMs we checked both model stability and collinearity of terms. We checked for model stability by comparing our models to those which excluded levels of the random effects one at a time. We checked for collinearity using Variance Inflation Factors (VIF, Field2005) using the function vif of the R-package car (Fox & Weisberg 2011). Output of these checks for final models reported in the main text are reported below for each model.

Stability:

Self-directed behaviours:

Playback phase:

|  | orig | min | max |
| --- | --- | --- | --- |
| (Intercept) | -3.81625 | -3.90438 | -3.72182 |
| conditionoutgroup | -0.05755 | -0.19419 | 0.069738 |
| trial_ | 0.050531 | -0.02682 | 0.169844 |
| conditionoutgroup:trial_ | 0.4398 | 0.342824 | 0.743315 |
| individual_@(Intercept) | 1.218282 | 1.137127 | 1.252446 |
| individual_@conditionoutgroup | 0.824177 | 0.720418 | 0.867418 |

Post phase:

|  | orig | min | max |
| --- | --- | --- | --- |
| (Intercept) | -4.00044 | -4.32318 | -3.88358 |
| conditionoutgroup | 0.141725 | -0.08068 | 0.524146 |
| trial_ | -0.30646 | -0.42489 | -0.15848 |
| individual_@(Intercept) | 1.245466 | 1.209804 | 1.31953 |
| individual_@conditionoutgroup | 1.071027 | 0.990765 | 1.168126 |
| individual_@trial_ | 0.782323 | 0.646133 | 0.875831 |
| individual_@conditionoutgroup:trial_ | 1.173508 | 0.908117 | 1.270964 |

Social grooming:

Playback phase:

|  | orig | min | max |
| --- | --- | --- | --- |
| (Intercept) | -4.04565 | -4.21342 | -3.83904 |
| conditionoutgroup | -0.67498 | -0.84223 | -0.52504 |
| trial_ | 0.382704 | 0.095325 | 0.526067 |
| conditionoutgroup:trial_ | 1.212603 | 1.057685 | 1.491264 |
| individual_@(Intercept) | 1.643965 | 1.600305 | 1.684373 |
| individual_@conditionoutgroup | 1.487359 | 1.378702 | 1.561016 |
| individual_@trial_ | 1.496738 | 1.393599 | 1.532922 |

Post phase:

|  | orig | min | max |
| --- | --- | --- | --- |
| (Intercept) | -2.22742 | -2.31868 | -2.05973 |
| conditionoutgroup | 0.356137 | 0.147173 | 0.478959 |
| trial_ | -0.12669 | -0.24152 | -0.06333 |
| conditionoutgroup:trial_ | 0.302189 | 0.200078 | 0.478127 |
| individual_@(Intercept) | 1.331321 | 1.260973 | 1.366647 |
| individual_@conditionoutgroup | 0.903586 | 0.744792 | 0.9402877 |

Rest:

Playback phase:

|  | orig | min | max |
| --- | --- | --- | --- |
| (Intercept) | 0.201345 | 0.10569 | 0.280049 |
| conditionoutgroup | -0.28897 | -0.39165 | -0.22492 |
| trial_ | -0.10938 | -0.16013 | -0.05337 |
| individual_@(Intercept) | 1.122841 | 1.061861 | 1.133948 |
| ividual_@conditionoutgroup | 0.952574 | 0.901543 | 0.964683 |
| individual_@trial_ | 0.985648 | 0.87538 | 1.004458 |
| _@conditionoutgroup:trial_ | 0.938642 | 0.8387 | 0.955592 |

Post phase:

|  | orig | min | max |
| --- | --- | --- | --- |
| (Intercept) | -0.39449 | -0.45803 | -0.31881 |
| conditionoutgroup | 0.084991 | 0.015986 | 0.145035 |
| trial_ | 0.006949 | -0.03834 | 0.046264 |
| individual_@(Intercept) | 1.048693 | 1.013839 | 1.06327 |
| individual_@conditionoutgroup | 0.842666 | 0.801032 | 0.856873 |
| individual_@trial_ | 0.841755 | 0.79308 | 0.856633 |
| individual_@conditionoutgroup:trial_ | 0.934645 | 0.887439 | 0.954468 |

Posture:

Playback phase:

|  | orig | min | max |
| --- | --- | --- | --- |
| (Intercept) | 0.03053 | -0.09379 | 0.113784 |
| conditionoutgroup | 0.934489 | 0.834449 | 1.134949 |
| trial_ | -0.37975 | -0.46276 | -0.28769 |
| individual_@(Intercept) | 1.154032 | 1.104104 | 1.177384 |
| individual_@conditionoutgroup | 1.155971 | 1.045225 | 1.189808 |
| individual_@trial_ | 0.965834 | 0.859399 | 0.989823 |
| individual_@conditionoutgroup:trial_ | 1.159146 | 0.978117 | 1.20363 |

Post phase:

|  | orig | min | max |
| --- | --- | --- | --- |
| (Intercept) | 0.785136 | 0.591745 | 0.917077 |
| conditionoutgroup | -0.80322 | -0.98073 | -0.6254 |
| trial_ | -0.30425 | -0.37771 | -0.22348 |
| individual_@(Intercept) | 1.535142 | 1.466322 | 1.573521 |
| ividual_@conditionoutgroup | 1.492244 | 1.421283 | 1.53445 |
| individual_@trial_ | 1.302583 | 1.244018 | 1.340888 |
| _@conditionoutgroup:trial_ | 1.396446 | 1.320193 | 1.442948 |

Aggression:

Playback phase:

|  | orig | min | max |
| --- | --- | --- | --- |
| (Intercept) | -2.71263 | -2.85 | -2.42636 |
| conditionoutgroup | 0.507166 | 0.377 | 0.63913 |
| trial_ | 0.056595 | -1.32E-19 | 0.136483 |
| individual@(Intercept) | 1.035238 | 1.02 | 1.066801 |

Post phase:

|  | orig | min | max |
| --- | --- | --- | --- |
| (Intercept) | -2.8645 | -3.0986 | -2.79109 |
| conditionoutgroup | -0.71432 | -1.0269 | -0.54518 |
| trial_ | 0.362144 | 0.254534 | 0.536614 |
| conditionoutgroup:trial_ | -0.92687 | -1.11609 | -0.76379 |
| individual@(Intercept) | 1.192609 | 1.105651 | 1.258793 |

Play:

Playback phase:

|  | orig | min | max |
| --- | --- | --- | --- |
| (Intercept) | -3.27746 | -3.47975 | -3.10598 |
| conditionoutgroup | -0.65295 | -0.88759 | -0.47055 |
| trial_ | 0.242178 | 0.134317 | 0.379453 |
| individual@(Intercept) | 1.260857 | 1.11128 | 1.319375 |

Post phase:

|  | orig | min | max |
| --- | --- | --- | --- |
| (Intercept) | -3.13491 | -3.47134 | -3.06255 |
| conditionoutgroup | -1.0808 | -1.37446 | -0.61336 |
| trial_ | 0.921859 | 0.830173 | 1.108717 |
| conditionoutgroup:trial_ | -1.44386 | -1.64313 | -1.34745 |
| individual@(Intercept) | 1.176242 | 1.112988 | 1.266259 |

Sex:

Playback phase:

|  | orig | min | max |
| --- | --- | --- | --- |
| (Intercept) | -3.63E+00 | -3.89911 | -3.43718 |
| conditionoutgroup | 4.87E-07 | -0.17013 | 0.16874 |
| trial_ | 8.48E-01 | 0.77534 | 1.002766 |
| individual@(Intercept) | 7.80E-01 | 0 | 0.90203 |

Post phase:

|  | orig | min | max |
| --- | --- | --- | --- |
| (Intercept) | -2.94837 | -3.27196 | -2.9168 |
| conditionoutgroup | -0.87718 | -1.29194 | -0.52617 |
| trial_ | 0.093649 | -0.05201 | 0.262164 |
| individual@(Intercept) | 0.000447 | 0 | 0.291807 |

Collinearity:

Self-directed behaviour:

Playback phase:

condition trial_ condition:trial_

1.004121 2.027535 2.029661

Post phase:

condition trial_

1.001308 1.001308

Social grooming:

Playback phase:

condition trial_ condition:trial_

1.004121 2.027535 2.029661

Post phase:

condition trial_ condition:trial_

1.003461 1.937691 1.935291

Rest:

Playback phase:

condition trial_

1.000169 1.000169

Post phase:

condition trial_

1.001308 1.001308

Posture:

Playback phase:

condition trial_

1.006567 1.006567

Post phase:

condition trial_

1.000737 1.000737

Aggression:

Playback phase:

condition trial_

1 1

Post phase:

condition trial_ condition:trial_

1 2 2

Play:

Playback phase:

condition trial_

1 1

Post phase:

condition trial_ condition:trial_

1 2 2

Sex:

Playback phase:

condition trial_

1 1

Post phase:

condition trial_

1 1
