# Supplementary material for: Increased alertness and moderate ingroup cohesion in bonobos’ response to outgroup cues
Source: PLoS One. 2024 Aug 21;19(8):e0307975. doi: 10.1371/journal.pone.0307975 (PMC11338468; doi:10.1371/journal.pone.0307975)
Supplement: S1 File — (ZIP) [file pone.0307975.s001.zip › Final/Supple_BonoboPlayback.docx]

**Graphs of significant interactions by condition and effect**


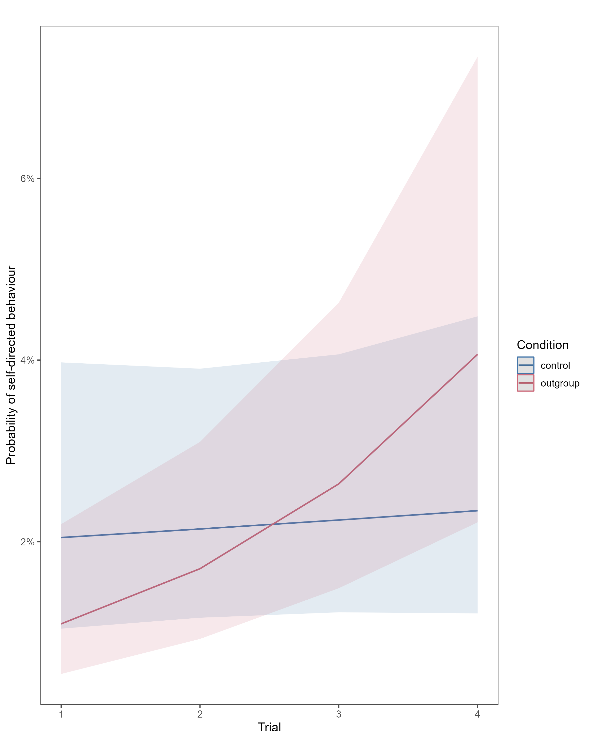

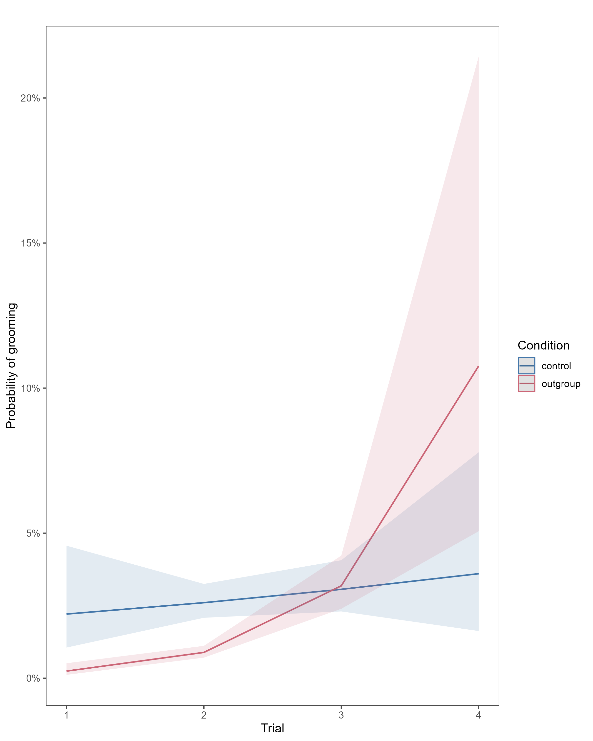


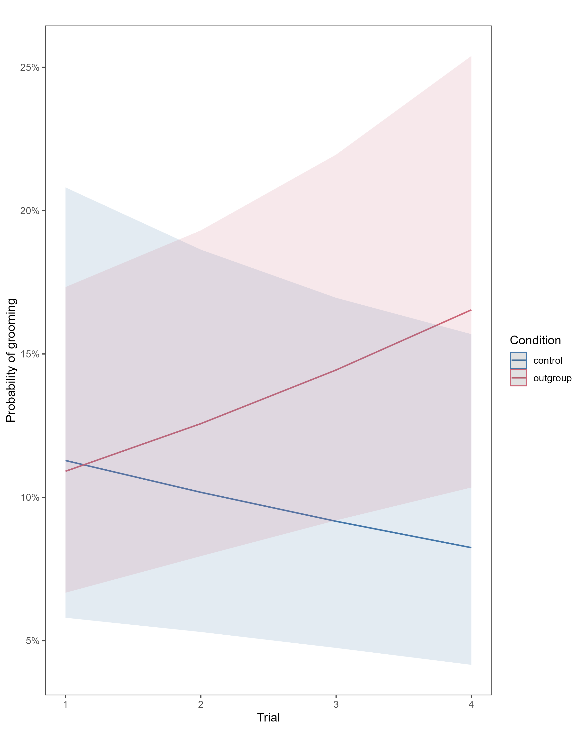

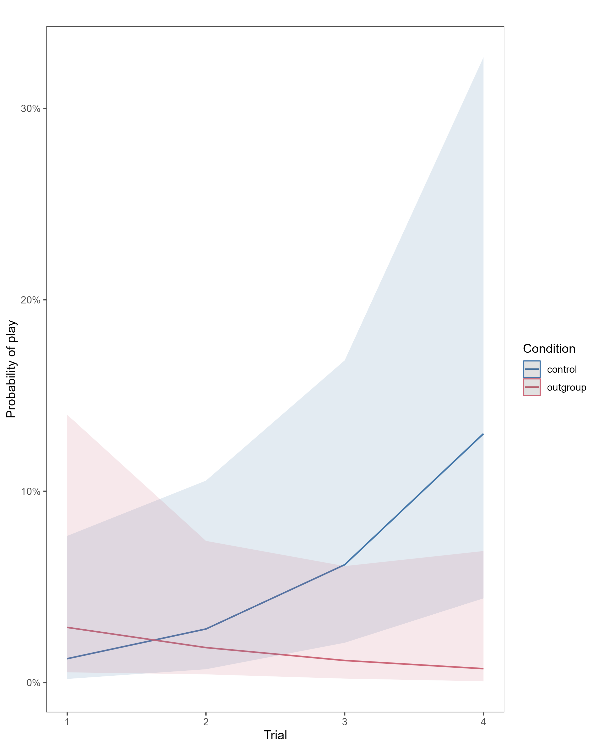


**Fig. S1.** Graphs of significant interactions between condition and trial. a) self-directed behaviour in playback phase b) grooming in playback phase c) grooming in post phase d) play in post phase. Daily variation is high, but both playback and controls trials were performed on the same day to control for such variation. The difference between conditions is what should be attended to.
