# Supplementary material for: Increased alertness and moderate ingroup cohesion in bonobos’ response to outgroup cues
Source: PLoS One. 2024 Aug 21;19(8):e0307975. doi: 10.1371/journal.pone.0307975 (PMC11338468; doi:10.1371/journal.pone.0307975)
Supplement: S1 File — (ZIP) [file pone.0307975.s001.zip › Final/SupportingModelResults.docx]

Maximal convergent singular models:

Singular models were overfitted and thus resulted in low significance for fixed terms compared to the final models presented in the main text. Singular models arise when the complexity of the model terms is more than can be supported by the data, and thus can result in low impact for any given variable of interest when included with many others. Model simplification is one strategy used to solve this, by removing the random effects and slopes with the least importance to the data until the model is no longer singular and can give meaningful results about the impact of each term on measures of interest. This strategy was taken in the main text, but for completeness we include singular models here.

Proximity: The models reported in the main text were the first to converge.

Behaviours: All behavioural models converged, and thus the results of the full (singular) models are shown here. The models included several predictor variables that overfit the data, and thus model simplification was necessary.

Self-directed behaviour:

Playback phase:

Model:

sd ~ condition * trial_ + (condition * trial_ + timeofday + time_ |

group_/individual_)

npar AIC LRT Pr(Chi)

<none> 1233.9

condition:trial_ 1 1232.8 0.82436 0.3639

Model:

sd ~ condition + trial_ + (condition * trial_ + timeofday + time_ |

group_/individual_)

npar AIC LRT Pr(Chi)

<none> 1232.8

condition 1 1230.8 0.00159 0.9682

trial_ 1 1232.5 1.74722 0.1862

Post phase:

Model:

sd ~ condition * trial_ + (condition * trial_ + timeofday + time_ |

group_/individual_)

npar AIC LRT Pr(Chi)

<none> 966.69

condition:trial_ 1 965.78 1.0927 0.2959

Model:

sd ~ condition + trial_ + (condition * trial_ + timeofday + time_ |

group_/individual_)

npar AIC LRT Pr(Chi)

<none> 965.78

condition 1 965.96 2.1747 0.1403

trial_ 1 965.46 1.6801 0.1949

Social grooming:

Playback phase:

Model:

grooming ~ condition * trial_ + (condition * trial_ + timeofday +

time_ | group_/individual_)

npar AIC LRT Pr(Chi)

<none> 1813.2

condition:trial_ 1 1811.2 0.0029771 0.9565

Model:

grooming ~ condition + trial_ + (condition * trial_ + timeofday +

time_ | group_/individual_)

npar AIC LRT Pr(Chi)

<none> 1811.2

condition 1 1812.1 2.8439 0.09172 .

trial_ 1 1812.2 2.9688 0.08488 .

Post phase:

Model:

grooming ~ condition * trial_ + (condition * trial_ + timeofday +

time_ | group_/individual_)

npar AIC LRT Pr(Chi)

<none> 1923.0

condition:trial_ 1 1921.1 0.13128 0.7171

Model:

grooming ~ condition + trial_ + (condition * trial_ + timeofday +

time_ | group_/individual_)

npar AIC LRT Pr(Chi)

<none> 1921.1

condition 1 1924.7 5.6000 0.01796 *

trial_ 1 1922.0 2.8585 0.09089 .

Rest:

Playback phase:

Model:

rest ~ condition * trial_ + (condition * trial_ + timeofday +

time_ | group_/individual_)

npar AIC LRT Pr(Chi)

<none> 4428.9

condition:trial_ 1 4433.2 6.3346 0.01184 *

Model:

rest ~ condition + trial_ + (condition * trial_ + timeofday +

time_ | group_/individual_)

npar AIC LRT Pr(Chi)

<none> 4433.2

condition 1 4431.2 -0.068347 1.0000

trial_ 1 4431.3 0.014723 0.9034

Post phase:

Model:

rest ~ condition * trial_ + (condition * trial_ + timeofday +

time_ | group_/individual_)

npar AIC LRT Pr(Chi)

<none> 3337.3

condition:trial_ 1 3335.6 0.24358 0.6216

Model:

rest ~ condition + trial_ + (condition * trial_ + timeofday +

time_ | group_/individual_)

npar AIC LRT Pr(Chi)

<none> 3335.6

condition 1 3333.6 0.025185 0.8739

trial_ 1 3333.9 0.285786 0.5929

Posture:

Playback phase:

Model:

sit ~ condition * trial_ + (condition * trial_ + timeofday +

time_ | group_/individual_)

npar AIC LRT Pr(Chi)

<none> 2171.5

condition:trial_ 1 2169.8 0.29821 0.585

Model:

sit ~ condition + trial_ + (condition * trial_ + timeofday +

time_ | group_/individual_)

npar AIC LRT Pr(Chi)

<none> 2169.8

condition 1 2169.3 1.51214 0.2188

trial_ 1 2168.0 0.16518 0.6844

Post phase:

Model:

sit ~ condition * trial_ + (condition * trial_ + timeofday +

time_ | group_/individual_)

npar AIC LRT Pr(Chi)

<none> 1240.1

condition:trial_ 1 1238.9 0.79674 0.3721

Model:

sit ~ condition + trial_ + (condition * trial_ + timeofday +

time_ | group_/individual_)

npar AIC LRT Pr(Chi)

<none> 1238.9

condition 1 1237.3 0.46791 0.4940

trial_ 1 1236.9 0.02213 0.8817

Aggression:

Playback phase:

Model:

ag ~ condition * trial_ + (condition * trial_ + timeofday | group/individual)

npar AIC LRT Pr(Chi)

<none> 193.88

condition:trial_ 1 192.60 0.72138 0.3957

Model:

ag ~ condition + trial_ + (condition * trial_ + timeofday | group/individual)

npar AIC LRT Pr(Chi)

<none> 192.60

condition 1 190.95 0.35089 0.5536

trial_ 1 193.11 2.50932 0.1132

Post phase:

Model:

ag ~ condition * trial_ + (condition * trial_ + timeofday | group/individual)

npar AIC LRT Pr(Chi)

<none> 117.96

condition:trial_ 1 117.46 1.4982 0.2209

Model:

ag ~ condition + trial_ + (condition * trial_ + timeofday | group/individual)

npar AIC LRT Pr(Chi)

<none> 117.46

condition 1 117.50 2.04165 0.153

trial_ 1 114.80 -0.65503 1.000

Play:

Playback phase:

Model:

play ~ condition * trial_ + (condition * trial_ + timeofday |

group/individual)

npar AIC LRT Pr(Chi)

<none> 226.53

condition:trial_ 1 224.55 0.023412 0.8784

Model:

play ~ condition + trial_ + (condition * trial_ + timeofday |

group/individual)

npar AIC LRT Pr(Chi)

<none> 224.55

condition 1 224.24 1.6953 0.1929

trial_ 1 223.88 1.3315 0.2485

Post phase:

Model:

play ~ condition * trial_ + (condition * trial_ + timeofday |

group/individual)

npar AIC LRT Pr(Chi)

<none> 167.41

condition:trial_ 1 165.52 0.11248 0.7373

Model:

play ~ condition + trial_ + (condition * trial_ + timeofday |

group/individual)

npar AIC LRT Pr(Chi)

<none> 165.52

condition 1 164.59 1.0781 0.2991

trial_ 1 163.93 0.4149 0.5195

Sex:

Playback phase:

Model:

sexd ~ condition * trial_ + (condition * trial_ + timeofday |

group/individual)

npar AIC LRT Pr(Chi)

<none> 130.07

condition:trial_ 1 143.09 15.026 0.000106 ***

Model:

sexd ~ condition + trial_ + (condition * trial_ + timeofday |

group/individual)

npar AIC LRT Pr(Chi)

<none> 143.09

condition 1 139.94 -1.1560 1

trial_ 1 134.84 -6.2565 1

Post phase:

Model:

sexd ~ condition * trial_ + (condition * trial_ + timeofday |

group/individual)

npar AIC LRT Pr(Chi)

<none> 94.893

condition:trial_ 1 95.182 2.2887 0.1303

Model:

sexd ~ condition + trial_ + (condition * trial_ + timeofday |

group/individual)

npar AIC LRT Pr(Chi)

<none> 95.182

condition 1 89.744 -3.4381 1

trial_ 1 91.269 -1.9135 1
