# Supplementary figures and images for: Increased alertness and moderate ingroup cohesion in bonobos’ response to outgroup cues
Source: PLoS One. 2024 Aug 21;19(8):e0307975. doi: 10.1371/journal.pone.0307975 (PMC11338468; doi:10.1371/journal.pone.0307975)

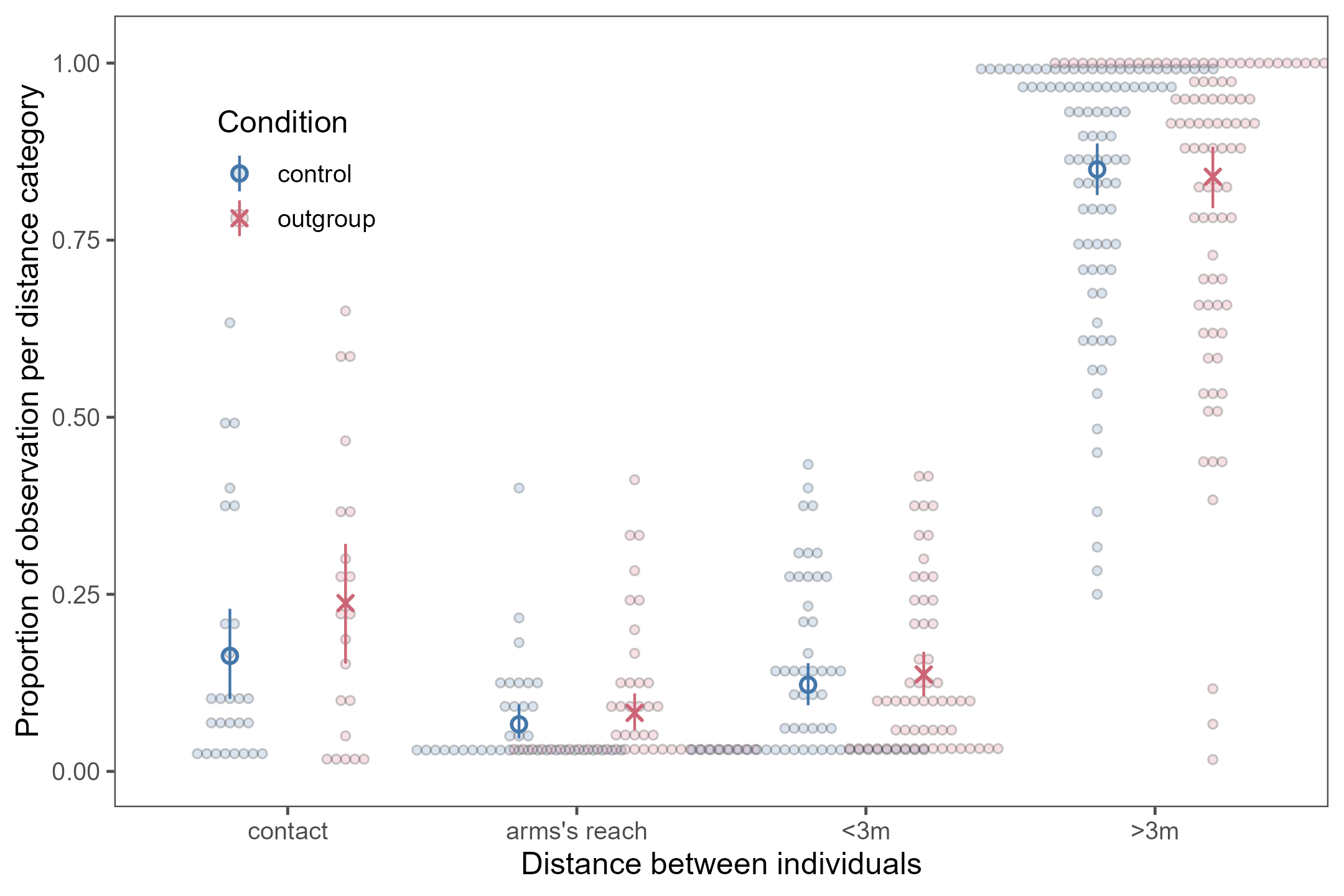

Supplement: S1 File — (ZIP) [file pone.0307975.s001.zip › Final/figures/Fig1a.tiff]

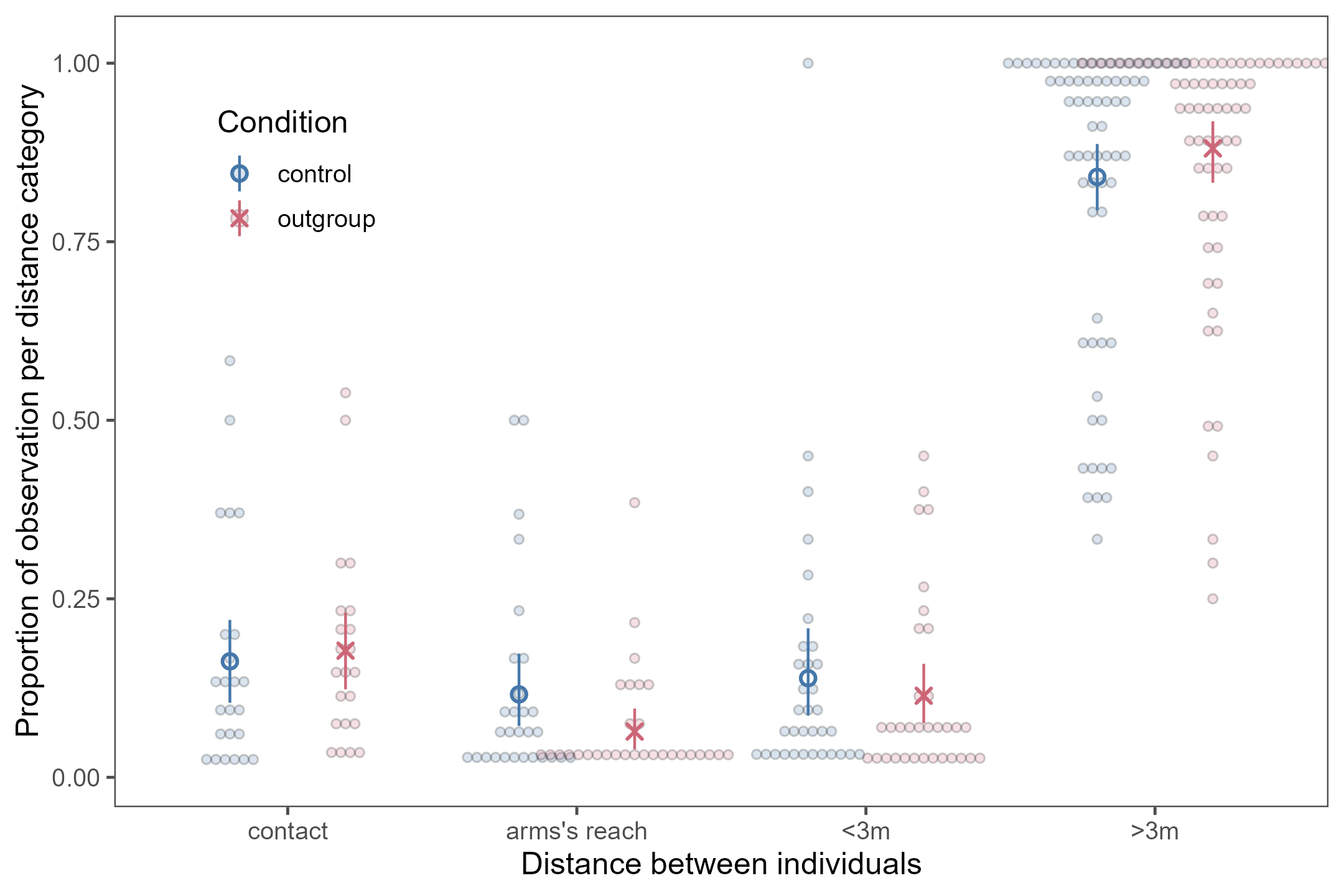

Supplement: S1 File — (ZIP) [file pone.0307975.s001.zip › Final/figures/Fig1b.tiff]

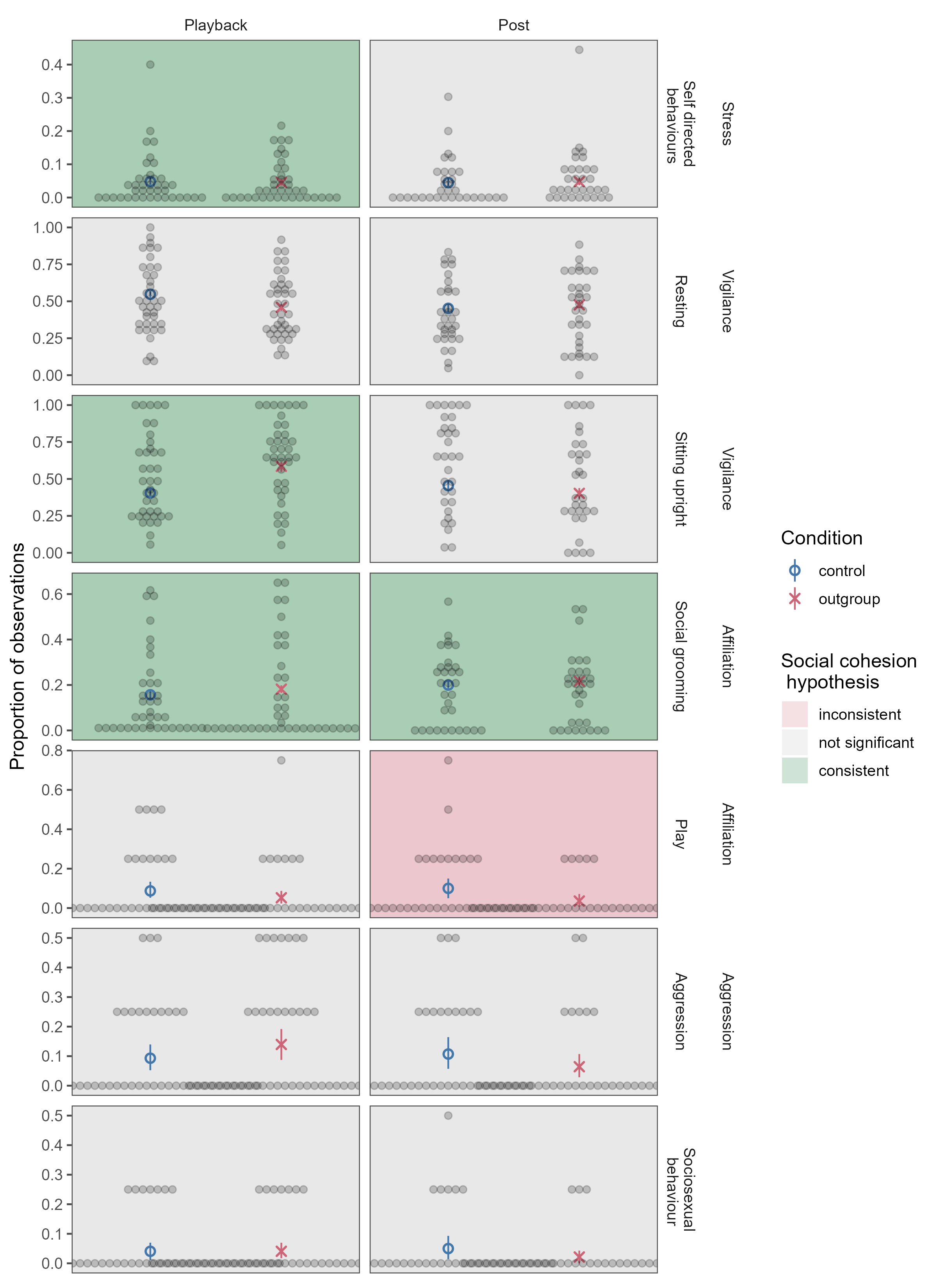

Supplement: S1 File — (ZIP) [file pone.0307975.s001.zip › Final/figures/Fig2.tiff]

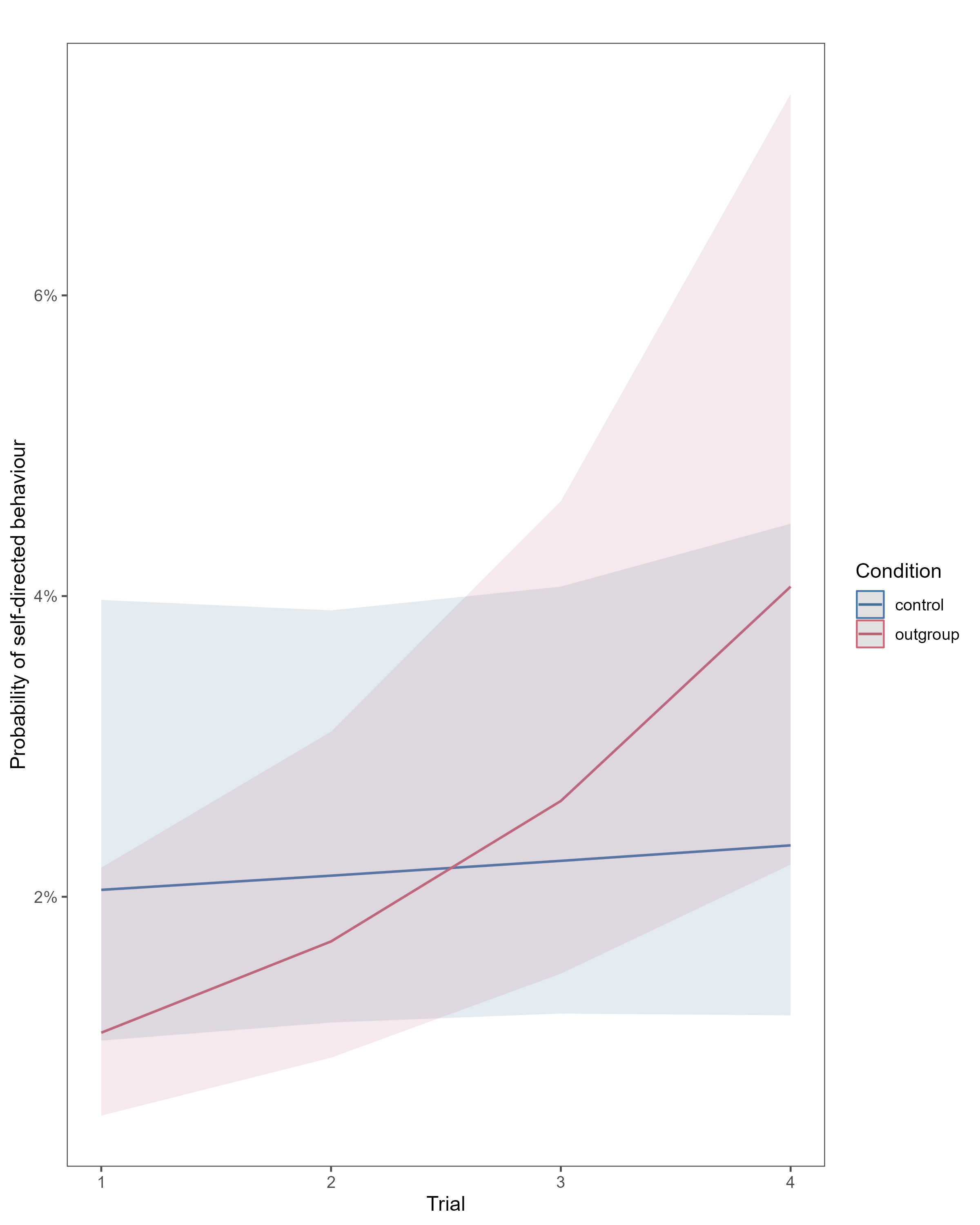

Supplement: S1 File — (ZIP) [file pone.0307975.s001.zip › Final/figures/FigS1a.tiff]

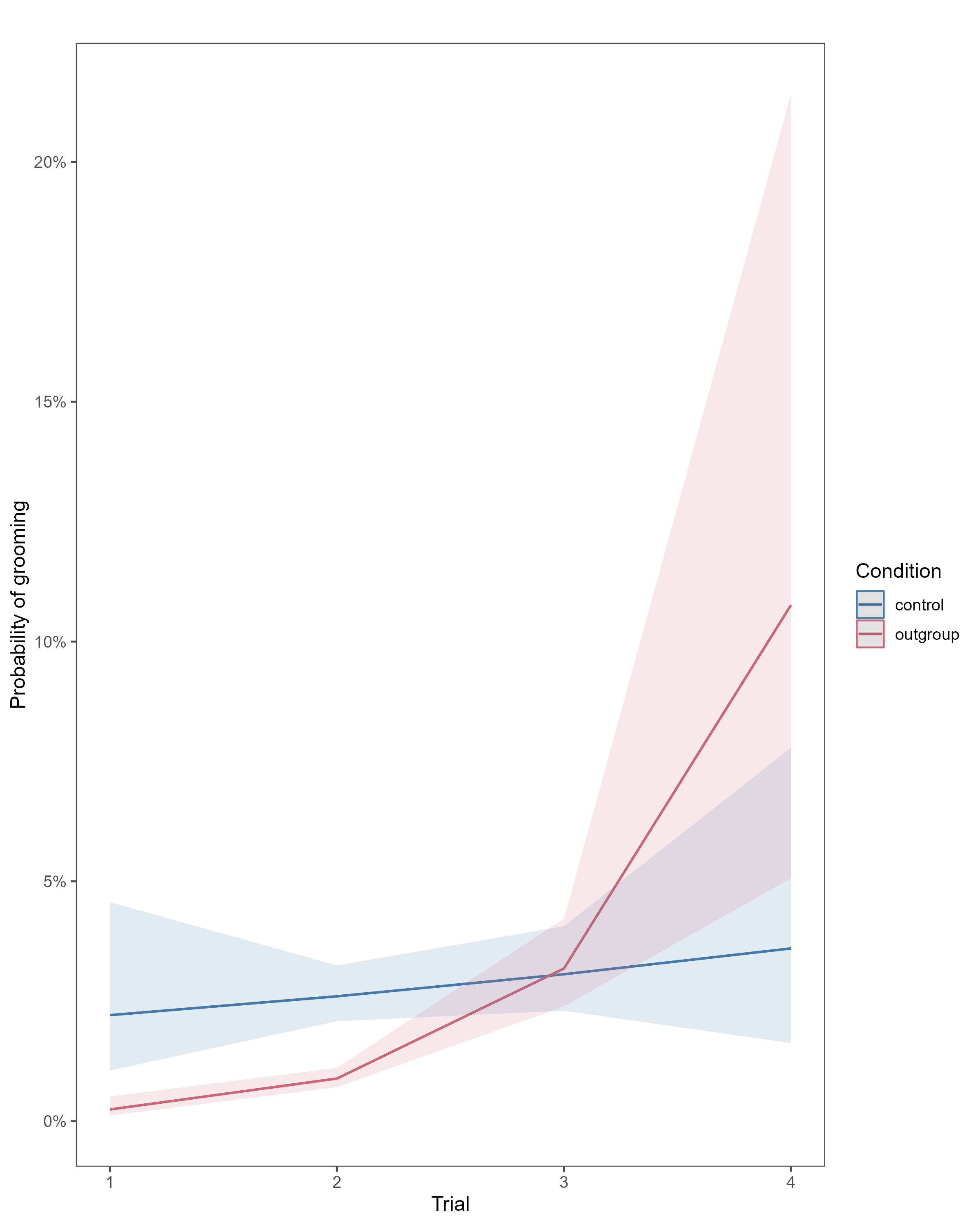

Supplement: S1 File — (ZIP) [file pone.0307975.s001.zip › Final/figures/FigS1b.tiff]

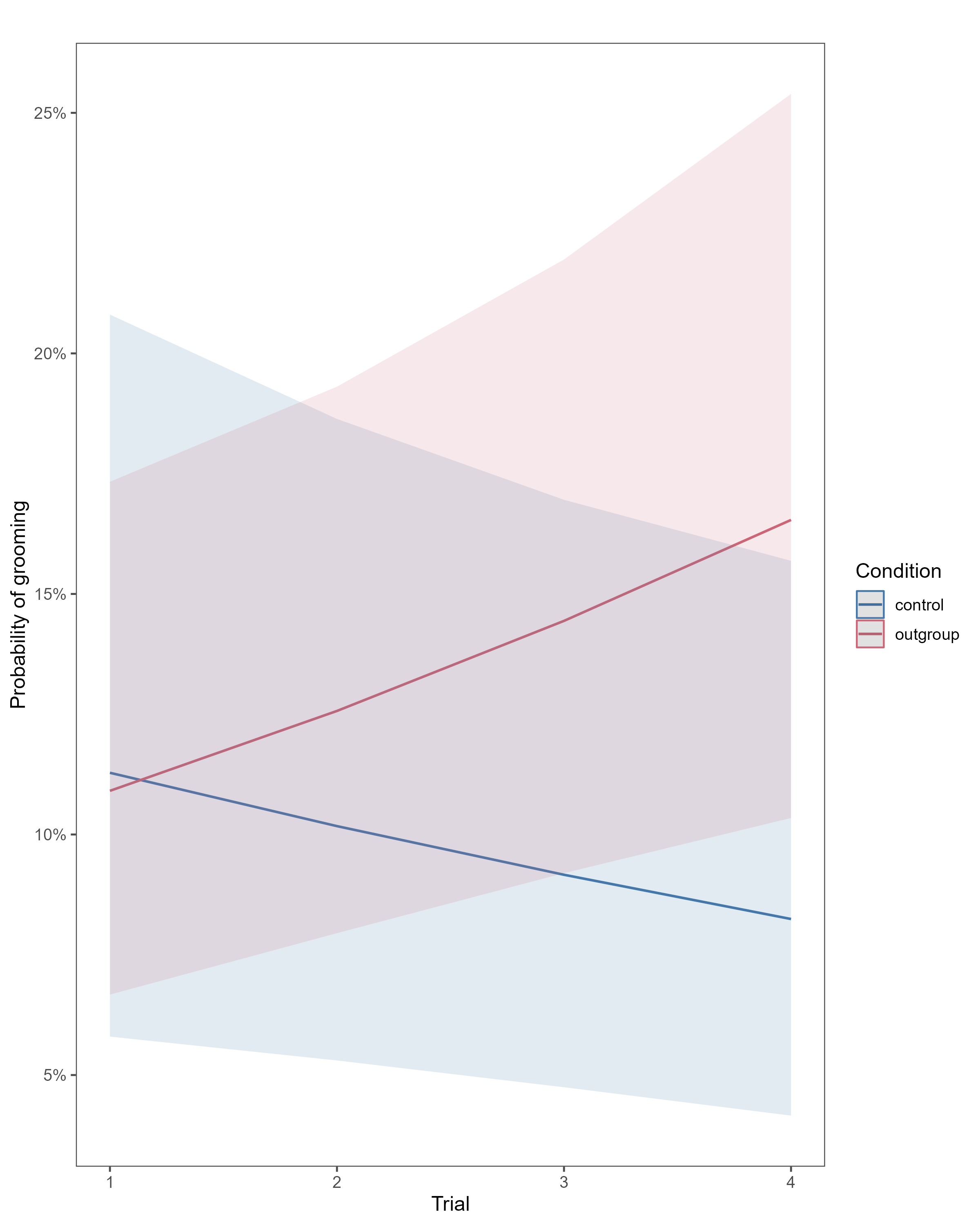

Supplement: S1 File — (ZIP) [file pone.0307975.s001.zip › Final/figures/FigS1c.tiff]

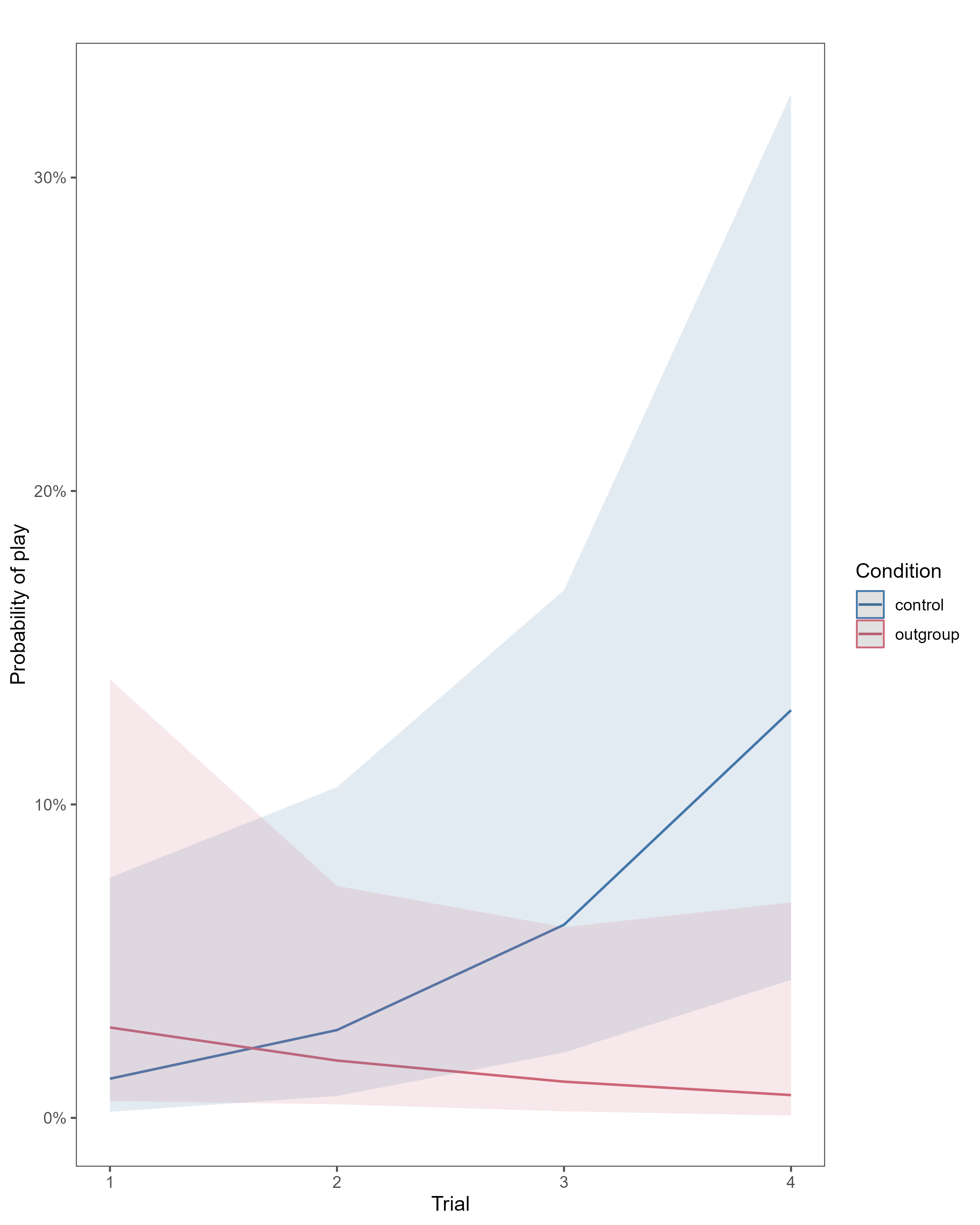

Supplement: S1 File — (ZIP) [file pone.0307975.s001.zip › Final/figures/FigS1d.tiff]
